# Retinoic acid promotes tissue vitamin A status and modulates adipose tissue metabolism of neonatal rats exposed to maternal high-fat diet-induced obesity

**DOI:** 10.1017/jns.2022.53

**Published:** 2022-07-08

**Authors:** Libo Tan, Yanqi Zhang, Hui Wang, Heleena Haberer

**Affiliations:** 1 Department of Human Nutrition, University of Alabama, 407 Russell Hall, 504 University Blvd, Tuscaloosa, AL 35487, USA; 2 Department of Biological Sciences, University of Alabama, Tuscaloosa, AL 35487, USA

**Keywords:** Adipose tissue, Maternal obesity, Neonate, Neonatal lung, Neonatal obesity, Retinoic acid, Vitamin A, BAT, brown adipose tissue, BW, body weight, HFD, high fat diet, LRAT, lecithin:retinol acyltransferase, NFD, normal fat diet, P, postnatal, RA, retinoic acid, RAR, retinoic acid receptor, RXR, retinoid X receptor, UCP, uncoupling protein, UPLC, ultra-high-performance liquid chromatography, VA, vitamin A, WAT, white adipose tissue

## Abstract

Maternal obesity may compromise the micronutrient status of the offspring. Vitamin A (VA) is an essential micronutrient during neonatal development. Its active metabolite, retinoic acid (RA), is a key regulator of VA homeostasis, which also regulates adipose tissue (AT) development in obese adults. However, its role on VA status and AT metabolism in neonates was unknown and it was determined in the present study. Pregnant Sprague-Dawley rats were randomised to a normal fat diet (NFD) or a high fat diet (HFD). From postnatal day 5 (P5) to P20, half of the HFD pups received oral RA every 3 d (HFDRA group). NFD pups and the remaining HFD pups (HFD group) received placebo. Six hours after dosing on P8, P14 and P20, *n* 4 pups per group were euthanised for different measures. It was found that total retinol concentration in neonatal liver and lung was significantly lower in the HFD group than the NFD group, while the concentrations were significantly increased in the HFDRA group. The HFD group exhibited significantly higher body weight (BW) gain, AT mass, serum leptin and adiponectin, and gene expression of these adipokines in white adipose tissue compared with the NFD group; these measures were significantly reduced in the HFDRA group. BAT UCP2 and UCP3 gene expression were significantly higher in pups receiving RA. In conclusion, repeated RA treatment during the suckling period improved the tissue VA status of neonates exposed to maternal obesity. RA also exerted a regulatory effect on neonatal obesity development by reducing BW gain and adiposity and modulating AT metabolism.

## Introduction

Presently, maternal overweight and obesity affect 48 % of pregnancies in the United States and 38⋅9 million women globally^(1,2)^. In addition, ~40 % of women in the United States gain an excessive amount of weight during pregnancy^(3)^. It has been well known that maternal obesity and excessive gestational weight gain may programme obesity in the offspring and result in adverse consequences for neonatal and long-term health and well-being, including the micronutrient status of the offspring^(4,5)^.

Vitamin A (VA, retinol) is a key micronutrient that is required during neonatal development for innate and adaptive immunity, haematopoiesis, and growth and differentiation of many types of cells^(6,7)^. VA-deficient infants have higher mortality and are at increased risk of infectious diseases^(8,9)^. In addition, VA is essential for normal postnatal development of the lung, among other crucial functions^(10,11)^. Significantly lower serum concentrations of retinol have been reported in adult obese humans and rodents compared with their normal-weight counterparts^(12–16)^. Our previous study showed that maternal diet-induced obesity was associated with a decreased serum concentration of retinol in neonatal rats^(17)^. Decreased VA concentrations in tissues, including the liver, adipose, lungs, pancreas and kidneys, were reported in diet-induced obese mice *v*. normal mice in two previous studies, leading the authors to conclude that, via an unknown mechanism, there was a tissue deficiency of VA associated with obesity^(18,19)^. Together, these evidence indicated potentially altered neonatal VA status and metabolism associated with maternal obesity.

Retinoic acid (RA), the active metabolite of VA, is known to play significant roles in VA homeostasis and status via regulating the expression of genes involved in VA metabolism. Its target genes include lecithin:retinol acyltransferase (LRAT) and RA hydroxylases of the CYP26 family of cytochrome P450 genes, which encode for the enzymes that catalyse the esterification of retinol for storage and the oxidation of RA, respectively^(20)^. The mRNA expression of LRAT and CYP26 were reported to be down-regulated in VA-deficient tissues, while the expression was rapidly up-regulated when RA was administered^(21,22)^. In neonatal rats, acute RA treatment was found to significantly increase the retinol uptake and esterification in the lung, and therefore its total retinol concentration^(23)^. Despite these previous findings and knowledge, the effect of RA on VA status of neonates in an obesogenic environment, however, has never been studied.

Meanwhile, RA has been reported to be a key regulator of adipose tissue (AT) development in adult obese models^(24)^. Previous research reported that the ‘machinery’ required for the molecular action of RA, including retinoic acid receptors (RARs) and retinoid X receptors (RXRs), are all expressed in the AT^(25)^. In adult rodents, RA was shown to inhibit adipogenesis and stimulate angiogenesis and apoptosis in white adipose tissue (WAT), and to increase the adaptive thermogenesis of brown adipose tissue (BAT) via regulating the expression of uncoupling proteins (UCPs)^(26–29)^. Our previous study indicated that supplementing the maternal diet with VA during lactation significantly reduced the adiposity and modulated serum adipokines and lipids in neonatal and weanling rats from dams consuming a high fat diet (HFD)^(30)^. However, no study has evaluated the effect of direct RA administration on the AT development of neonates affected by maternal diet-induced obesity.

Therefore, the present study had a 2-fold objective. The primary aim was to determine the effects of oral RA treatments on VA status of rat offspring exposed to maternal diet-induced obesity. It was hypothesised that RA would increase VA concentrations that were reduced by maternal HFD consumption in key neonatal organs. The secondary aim was to assess the effects of RA on the adiposity and AT metabolism of the neonates. We hypothesised that RA would reduce the body weight (BW) gain and adiposity of neonatal rats exposed to maternal HFD consumption, with an influence on the adipokines and lipids profile. As a single oral dose of RA was reported in previous research to have a transient effect on the metabolism of neonatal rats, RA was administered for repeated times for a potentially sustained effect^(23)^.

## Materials and methods

### Animal experiment

The procedure for this experiment was approved by the Institutional Animal Care and Use Committee of the University of Alabama. Five pregnant Sprague-Dawley rats were purchased from Charles River Laboratories (Wilmington, MA, USA) and arrived on their second day of gestation. Rats were housed individually with a 12 h light/dark cycle with free access to food and water. After a 3-d acclimation, rats were randomised to either a normal fat diet (NFD = 25 % kcal from fat) or an HFD (50 % kcal from fat) both with a marginal level of VA at 0⋅35 retinol equivalents/kg. The diets were purchased from Research Diets, Inc (New Brunswick, NJ, USA) (Table 1). After delivery, half of the pups delivered by HFD mothers received oral all-*trans*-RA (Sigma-Aldrich, MO, USA) treatments, while the other half and pups of the NFD mothers received canola oil as placebo. The three groups of pups (*n* 12 per group) were designated as NFD, HFD and HFDRA, respectively.

**Table 1.** Diet composition for Sprague-Dawley rats fed normal or high-fat purified diet

Ingredient	Normal fat diet^a^		High fat diet^b^	
	g	kcal	g	kcal
Casein	200	800	200	800
l-Cystine	3	12	3	12
Corn starch	353⋅8	1415	101⋅2	405
Maltodextrin	125	500	125	500
Sucrose	68⋅8	275	68⋅8	275
Cellulose	50	0	50	0
Soyabean oil	25	225	25	225
Lard	87⋅7	789	200	1800
Mineral mix, S10026	10	0	10	0
Dicalcium phosphate	13	0	13	0
Calcium carbonate	5⋅5	0	5⋅5	0
Potassium citrate	16⋅5	0	16⋅5	0
Vitamin mix, V10001	10	40	10	40
Choline bitartrate	2	0	2	0
Food colour	0⋅05	0	0⋅05	0
Total				
	g%	kcal%	g%	kcal%
Fat	12	25	27	50
Carbohydrate	57	55	37	30
Protein	21	20	24	20
Total	−	100	−	100
kcal/g	4⋅2	−	4⋅9	−

Diet composition for Sprague-Dawley rats fed normal or high-fat purified diets. Formulation details are provided in grams, g, and kilocalories, kcal.

a Research diets (rodent diet with 25 % kcal fat, D18100206).

b Research diets (rodent diet with 50 % kcal fat, D18100207).

**Fig. 1.**
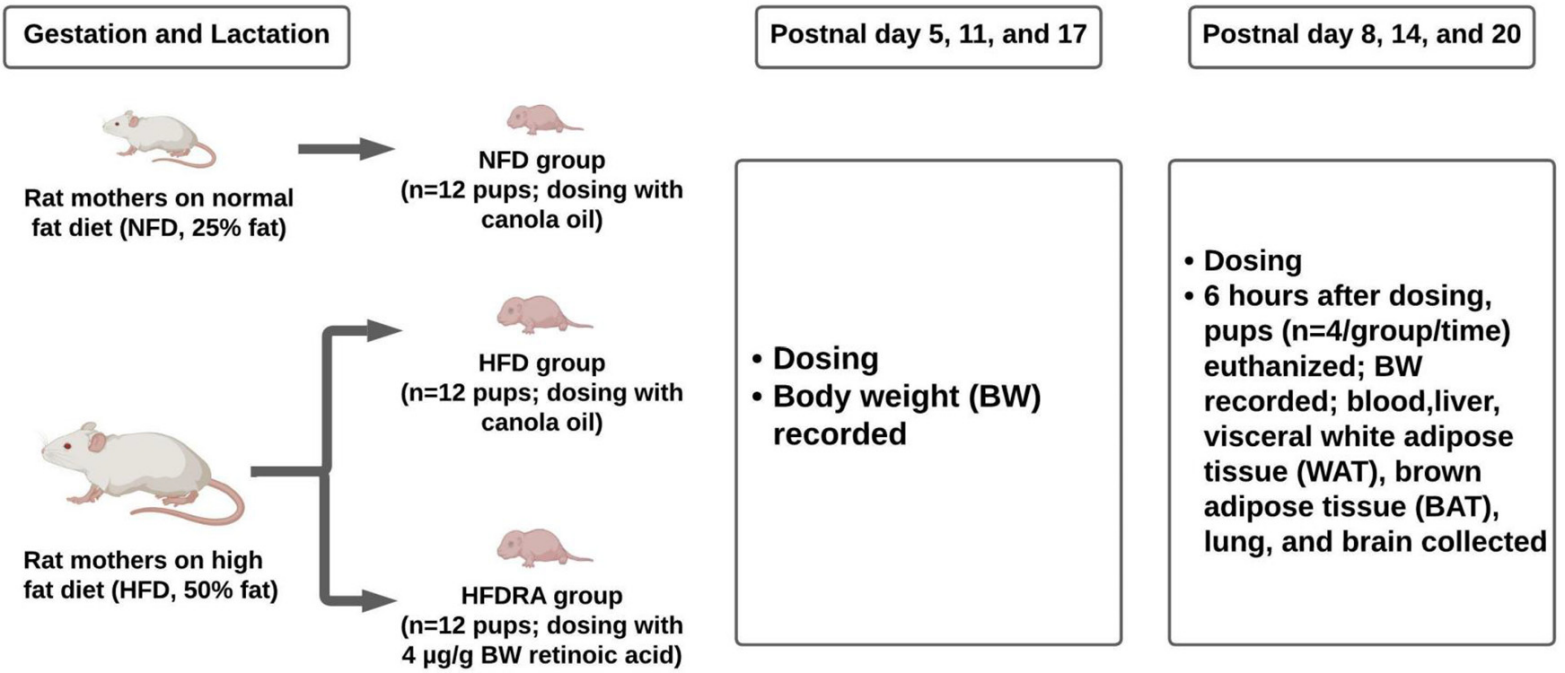
Schematic diagram of experimental procedures.

The schematic diagram of the study design is shown in Fig. 1. On postnatal day 5 (P5) and P8, respectively, HFDRA pups received an oral RA dose via feeding pipette at 4 μg/g BW. HFD pups and NFD pups both received canola oil at the same dosage. Six hours after the dose administration on P8, *n* 4 pups per group were euthanised. Blood, liver, visceral WAT (WAT surrounding the intra-abdominal organs), interscapular BAT, lung and brain were collected. On P11 and P14, remaining pups in each group (*n* 8 per group) received their respective treatment. Six hours after the administration on P14, *n* 4 pups per group were euthanised for tissue collection. Similar dosing and euthanisation procedures were conducted on P17 and P20. To sum up, pups euthanised at P8, P14 and P20 received two, four and six doses of RA, respectively. Pups’ BW and the weights of WAT and BAT were recorded. Pups’ BW gain was calculated as the BW at the euthanisation time minus that at P4.

### Serum and tissue analysis

#### Serum and tissue total retinol concentration

The concentration of total retinol (esterified + unesterified retinol) in serum, liver, lung, WAT, BAT and brain was analysed by ultra-high-performance liquid chromatography (UPLC) with a photodiode array detector and HSS T3 (1⋅8 μm, 2⋅1 mm × 100 mm) column (Acquity UPLC System; Waters, Milford, MA) following our previous method^(17)^. Briefly, 100 μl of serum sample or 0⋅1 g of tissue sample was added to or homogenised with 1⋅9 ml of ethanol and incubated at room temperature for 1 h. Saponification was achieved by adding 100 μl potassium hydroxide and 100 μl of 20 % pyrogallol to samples and being incubated in 55°C water bath for 30 min. After cooling down, 4 ml of hexane (with 0⋅1 % butylated hydroxytoluene) and 2 ml of dd H_2_O were added. After centrifugation for 15 min, the upper phase was collected, an internal standard (retinyl acetate, Sigma-Aldrich, St. Louis, MO) was added, and the solvent was dried under nitrogen. The dried sample was rinsed by hexane and reconstituted with 100 μl of acetonitrile:methanol (85:15, v/v). Possible precipitation was removed by centrifugation. Ten microlitres of the final sample was injected onto the HSS T3 column for analysis.

#### Serum lipids and adipokines

Serum samples from P14 and P20 were analysed for concentrations of lipids and adipokines. Serum samples from P8 were not adequate for the analyses. Concentrations of total cholesterol, triglycerides, HDL-C and LDC-C were measured using a Stanbio Sirus analyzer. Adiponectin concentration was assessed using a Millipore Rat Adiponectin ELISA (Billerica, MA) and leptin was measured using a Millipore Rat Leptin ELISA (Billerica, MA).

#### Leptin, Adiponectin and UCPs mRNA expression

For the mRNA determination of leptin and adiponectin in the WAT and that of UCP1, UCP2 and UCP3 in the BAT, samples from P20 were used, but not those from P8 or P14 due to inadequate tissue amount. Total RNA was extracted from tissue samples using Trizol (Invitrogen, Waltham, MA) and cDNA was prepared by using cDNA synthesis kit (QuantaBio, Beverly, MA). The equivalent of 1 μg RNA, as cDNA, was used for real-time qPCR analysis. The primers designed to detect the mRNA expression were as follows: Leptin (NM_013076.3), 5′-TCTCCGAGACCTCCTCCATCT-3′ (forward), and 5′-TTCCAGGACGCCATCCAG-3′ (reverse); Adiponectin (NM_144744.3), 5′-AAAATGTGGACCAGGCCTCT-3′ (forward) and 5′-TTGTCCCCTTCCCCATACAC-3′ (reverse); UCP-1 (NM_012682.2), 5′-AGAAGGATTGCCGAAACTGTAC-3′ (forward) and 5′-AGATCTTGCTTCCCAAAGAGG-3′ (reverse); UCP-2 (NM_019354.3), 5′-CCACAGCCACCGTGAAGTT-3′ (forward) and 5′-CGGACTTTGGCGGTGTCTA-3′ (reverse); UCP-3 (NM_013167.2), 5′-TGCTGAGATGGTGACCTACG-3′ (forward) and 5′-AGTGACAGGGGAAGTTGTCAG-3′ (reverse). β-actin was used as the housekeeping gene. The 2^−ΔΔCT^ method was used to compare the relative mRNA expression among groups^(31)^.

### Statistical analysis

Data are reported as means ± standard error of mean (sem). Differences among groups at each sampling time, *P*-value < 0⋅05, were determined by using one-way ANOVA followed by Bonferroni post-test in GraphPad Prism software (San Diego, CA, USA).

## Results

### Body weight and adiposity

**Fig. 2.**
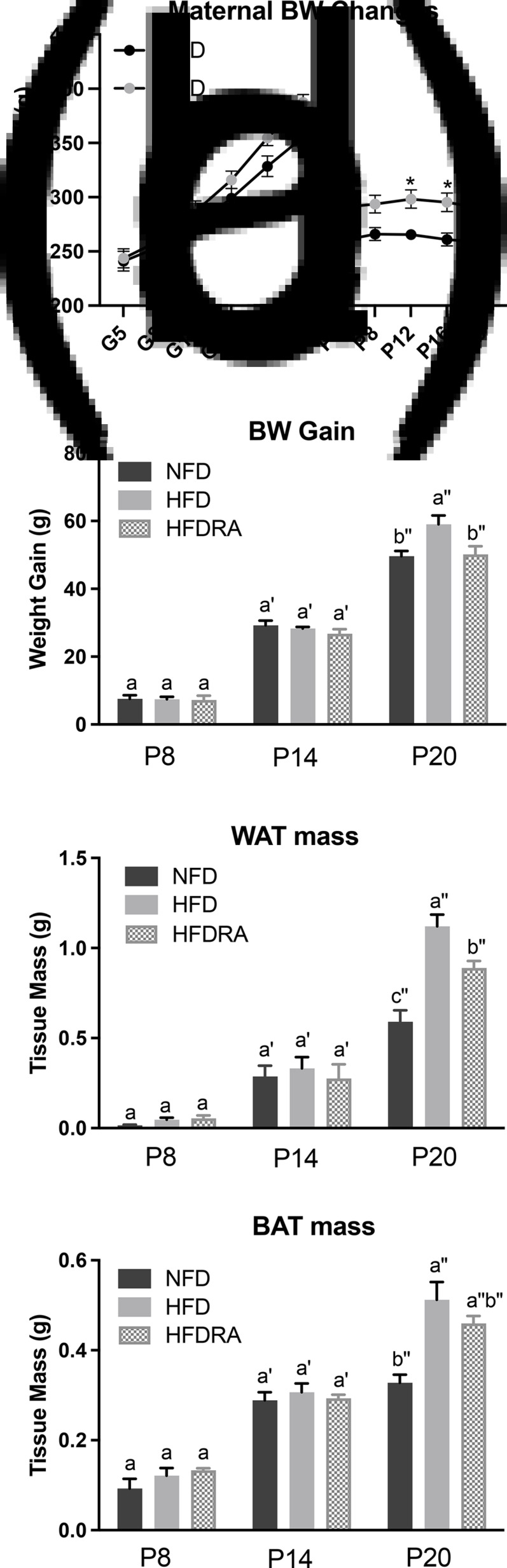
Maternal body weight from gestational day 5 to postnatal day 20 (a), the body weight gain (b), visceral white adipose tissue mass (c) and brown adipose tissue mass (d) of rat pups at postnatal day 8, postnatal day 14 and postnatal day 20. The body weight gain of rat pups was calculated as the body weight at the given time point minus that at postnatal day 4. Bars show means ± sem. The maternal body weight was compared between NFD and HFD at individual time points using Student's *t* test; significant differences were indicated by *, *n* 2 or 3 per group, *P* < 0⋅05. The body weight gain and mass of adipose tissues of rat pups were compared between different groups at individual time points using one-way ANOVA followed by Bonferroni post-test; significant differences were indicated by different letters, a″ > b″ > c″, *n* 4 per group, *P* < 0⋅05.

A significantly higher BW in rat mothers consuming the HFD compared with those fed the NFD was noted from P12 till the end of the study (*P* < 0⋅05; Fig. 2(a)). As shown in Fig. 2(b)–(d), at P8 and P14, no significant difference in BW gain, WAT mass and BAT mass of pups was noted among groups. At P20, all three measurements were significantly higher in the HFD group than in the NFD group (BW gain: 59⋅05 ± 2⋅57 g *v*. 49⋅63 ± 1⋅54 g, *P* < 0⋅001; WAT mass: 1⋅03 ± 0⋅10 g *v*. 0⋅59 ± 0⋅06 g, *P* < 0⋅0001; BAT mass: 0⋅51 ± 0⋅04 g *v*. 0⋅33 ± 0⋅02 g, *P* < 0⋅0001), confirming that maternal HFD consumption during gestation and lactation could result in a significantly higher BW gain and excessive adiposity in the young offspring. At P20, both the BW gain and the WAT mass were significantly decreased in the HFDRA group compared with the HFD group (BW gain: 50⋅15 ± 2⋅42 g *v*. 59⋅05 ± 2⋅57 g, *P* < 0⋅01; WAT mass: 0⋅89 ± 0⋅04 g *v*. 1⋅03 ± 0⋅10 g, *P* < 0⋅05), showing the effects of RA treatments on slowing the BW gain and reducing the adiposity. There was no significant difference noted in the BAT mass between the HFDRA and the HFD group (0⋅46 ± 0⋅02 g *v*. 0⋅51 ± 0⋅04 g, *P* > 0⋅05).

### Serum and tissue vitamin A

**Fig. 3.**
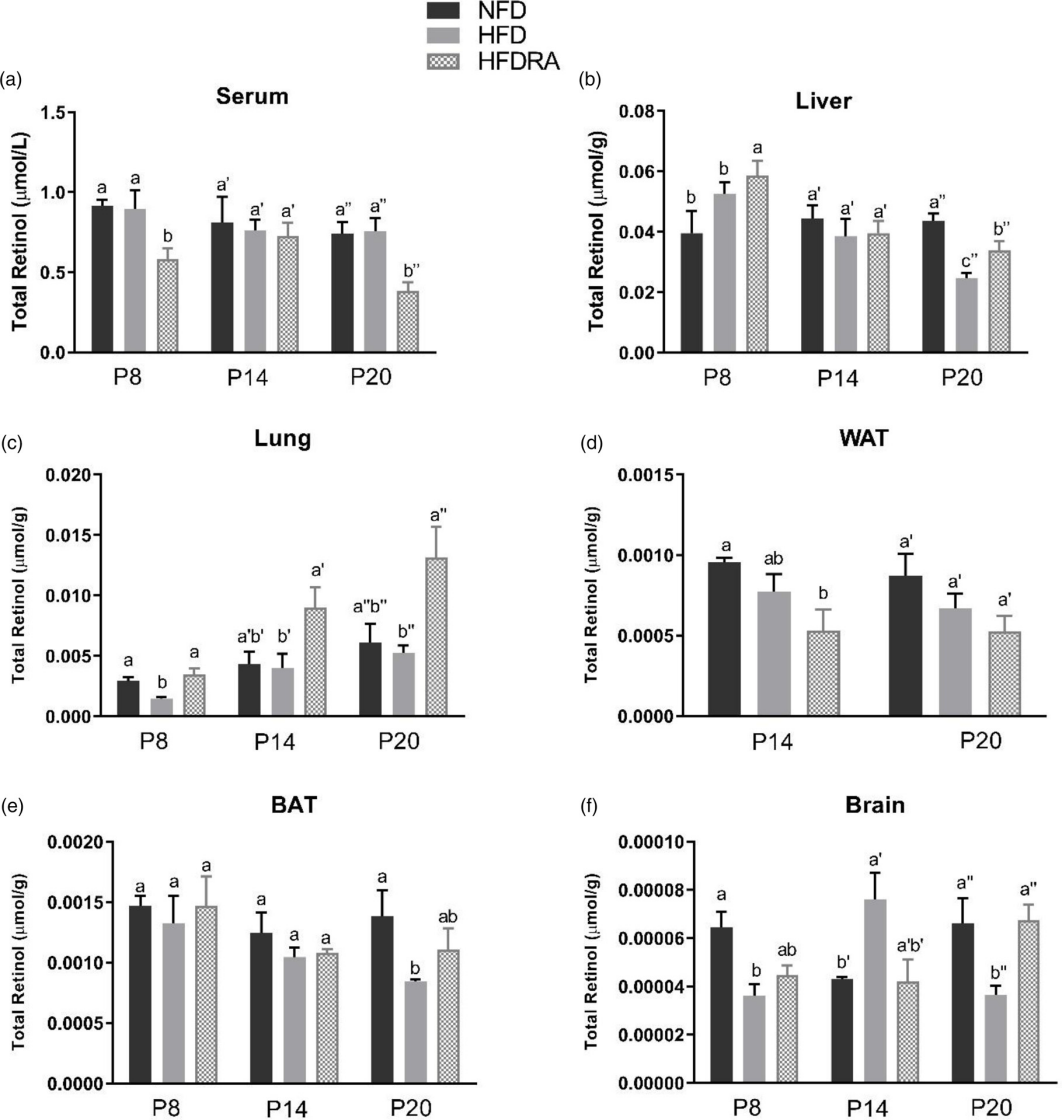
Concentrations of total retinol in serum (a), liver (b), lung (c), visceral white adipose tissue (d), brown adipose tissue (e) and brain (f) of rat pups at postnatal day 8, postnatal day 14 and postnatal day 20. Bars show means ± sem, *n* 4 per group. One-way ANOVA followed by a Bonferroni post-test was conducted at individual time point. Different letters at each time point indicate statistically significant differences, a > b, a′ > b′, a″ > b″ > c″, *P* < 0⋅05. *Note:* visceral white adipose tissue sample collected at P8 was not adequate for the analysis, and therefore the data are missing.

The total retinol concentration in pups’ serum, liver, lung, WAT, BAT and brain are shown in Fig. 3. Comparing between the NFD and the HFD group, the latter exhibited a significantly lower total retinol concentration in the liver at P20 (0⋅025 ± 0⋅002 μmol/g *v*. 0⋅040 ± 0⋅002 μmol/g, *P* < 0⋅05), in the lung at P8 (0⋅0014 ± 0⋅0002 μmol/g *v*. 0⋅0029 ± 0⋅0003 μmol/g, *P* < 0⋅01), in the BAT at P20 (0⋅0008 ± 0⋅00002 μmol/g *v*. 0⋅0014 ± 0⋅0002 μmol/g, *P* < 0⋅05) and in the brain at P8 and P20 (P8: 0⋅00003 ± 0⋅000005 μmol/g *v*. 0⋅00006 ± 0⋅000007 μmol/g, *P* < 0⋅05; P20: 0⋅00004 ± 0⋅000004 μmol/g *v*. 0⋅00007 ± 0⋅00001 μmol/g, *P* < 0⋅05).

The following differences were observed when comparing the HFD and the HFDRA group. At both P8 and P20, the serum total retinol was significantly lower in the HFDRA than the HFD group (P8: 0⋅58 ± 0⋅07 μmol/l *v*. 0⋅90 ± 0⋅12 μmol/l, *P* < 0⋅05; P20: 0⋅38 ± 0⋅06 μmol/l *v*. 0⋅730 ± 0⋅08 μmol/l, *P* < 0⋅05), while the liver total retinol was significantly higher in the HFDRA group (P8: 0⋅060 ± 0⋅005 μmol/g *v*. 0⋅050 ± 0⋅004 μmol/g, *P* < 0⋅05; P20: 0⋅034 ± 0⋅003 μmol/g *v*. 0⋅025 ± 0⋅002 μmol/g, *P* < 0⋅05). RA treatment also significantly increased the total retinol concentration in the lung at all three sampling times (P8: 0⋅0035 ± 0⋅0005 μmol/g *v*. 0⋅0014 ± 0⋅0002 μmol/g, *P* < 0⋅05; P14: 0⋅0090 ± 0⋅0017 μmol/g *v*. 0⋅0041 ± 0⋅0012 μmol/g, *P* < 0⋅05; P20: 0⋅013 ± 0⋅0025 μmol/g *v*. 0⋅0053 ± 0⋅0006 μmol/g, *P* < 0⋅05) and that in the brain at P20 (0⋅00007 ± 0⋅000006 μmol/g *v*. 0⋅00004 ± 0⋅000004 μmol/g, *P* < 0⋅01), restoring the concentrations to those in the NFD group.

### Serum leptin and adiponectin

**Fig. 4.**
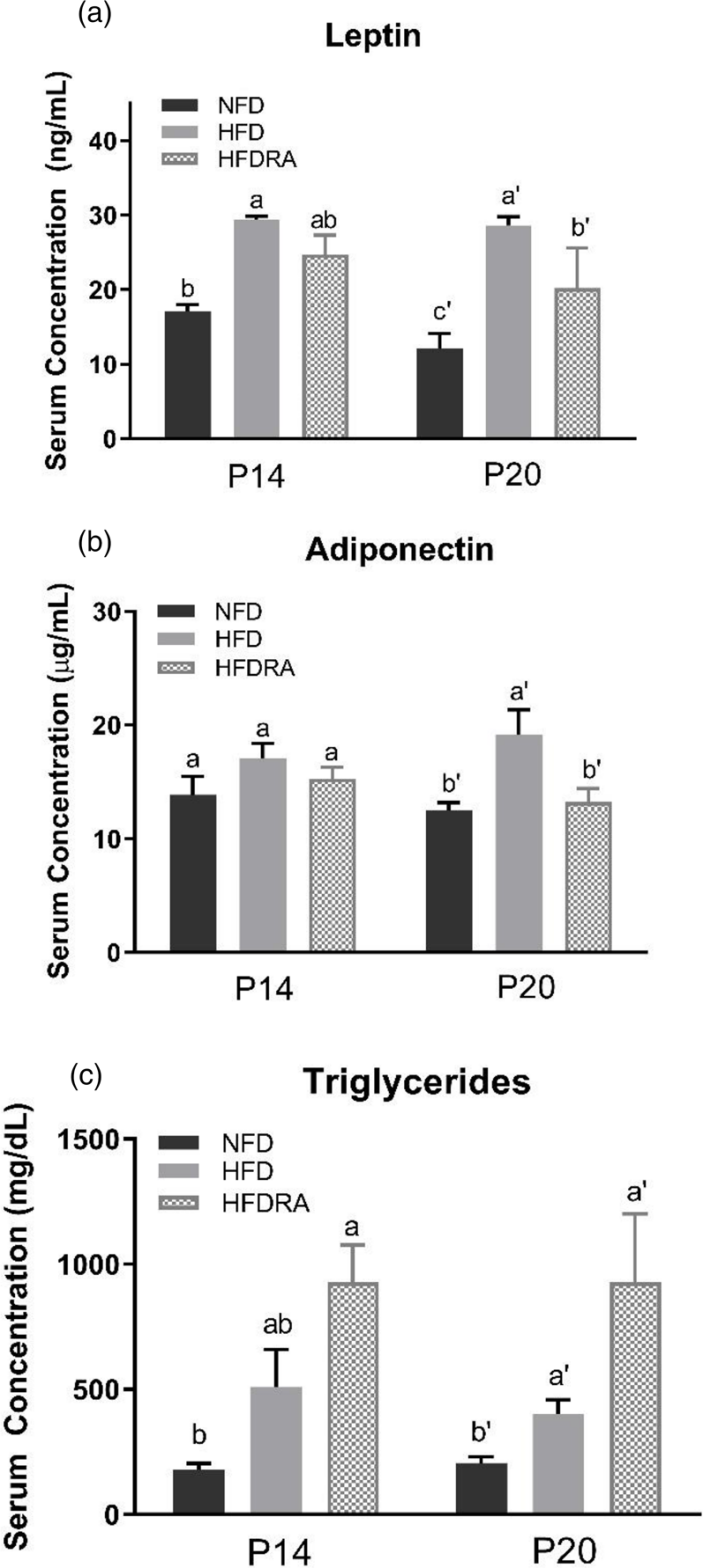
Serum leptin (a), adiponectin (b) and triglycerides (c) concentrations of rat pups at postnatal day 14 and postnatal day 20. Bars show means ± sem, *n* 4 per group. One-way ANOVA followed by a Bonferroni post-test was conducted at individual time point. Different letters at each time point indicate statistically significant differences, a > b, a′ > b′ > c′, *P* < 0⋅05.

At P20, serum leptin and adiponectin concentrations (Fig. 4(a), (b)) were both significantly higher in the HFD than the NFD group (leptin: 28⋅64 ± 1⋅15 ng/ml *v*. 12⋅14 ± 1⋅99 ng/ml, *P* < 0⋅0001; adiponectin: 19⋅11 ± 2⋅24 μg/ml *v*. 12⋅54 ± 0⋅62 μg/ml, *P* < 0⋅05). The comparison between the HFDRA and the HFD group indicated that RA treatment significantly reduced the concentrations of both (leptin: 20⋅18 ± 2⋅70 ng/ml *v*. 28⋅64 ± 1⋅15 ng/ml, *P* < 0⋅01; adiponectin: 15⋅67 ± 2⋅59 μg/ml *v*. 19⋅11 ± 2⋅24 μg/ml, *P* < 0⋅05). The pattern of changes in serum leptin was also observed at P14.

### Serum lipids

At P20, serum triglycerides concentration (Fig. 4(c)) was found to be significantly higher in the HFD group than the NFD group (400⋅75 ± 57⋅56 mg/dl *v*. 206 ± 24⋅46 mg/dl, *P* < 0⋅05). The concentration was even higher in the HFDRA group as compared with the HFD group (1104 ± 297 mg/dl *v*. 400⋅75 ± 57⋅56 mg/dl, *P* < 0⋅05). A similar trend was noted at P14, but the difference between the HFDRA and the HFD group did not reach statistical significance. There was no significant difference in serum total cholesterol, HDL-C and LDL-C observed among groups (data not shown).

### Leptin and adiponectin mRNA expression in the WAT

**Fig. 5.**
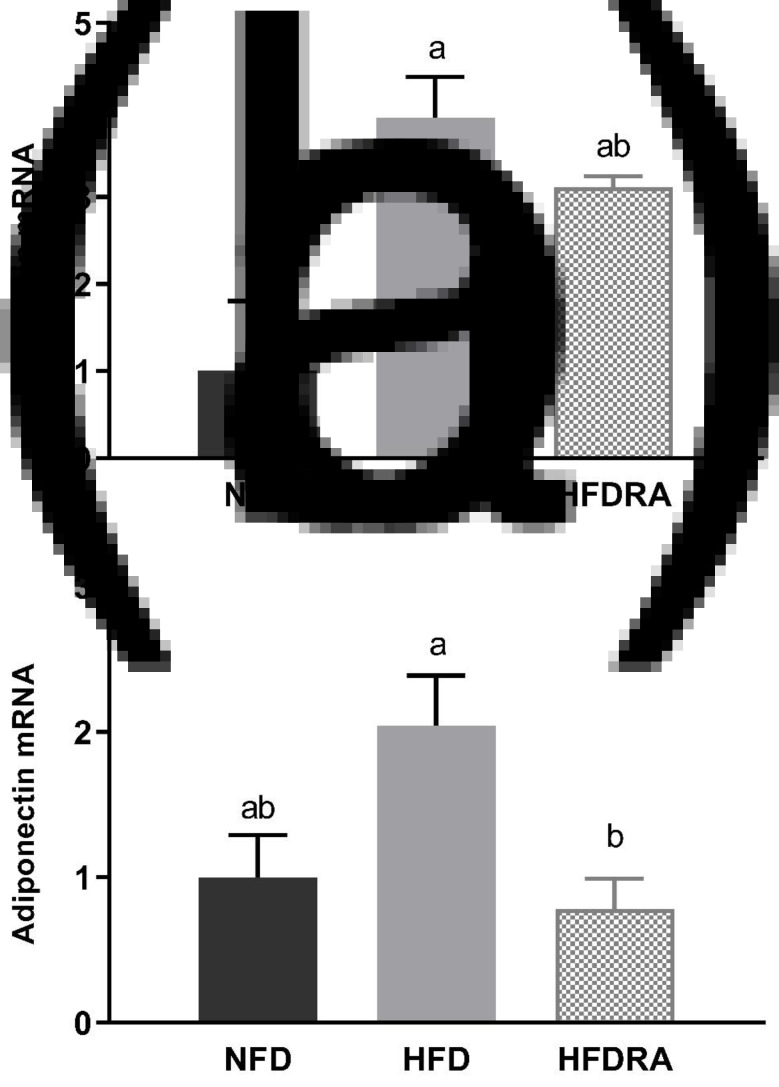
Visceral white adipose tissue leptin (a) and adiponectin (b) mRNA expression in rat pups at postnatal day 20. Results were normalised to β-actin mRNA. Bars show means ± sem, *n* 4 per group. One-way ANOVA followed by a Bonferroni post-test was conducted. Different letters indicate statistically significant differences, a > b, *P* < 0⋅05.

Consistently with the results on serum leptin and adiponectin, the leptin and adiponectin mRNA expression in WAT at P20 (Fig. 5) were both higher in the HFD group than in the NFD group, although it did not reach statistical significance for adiponectin expression. The HFDRA group showed a reduced trend of leptin mRNA expression while exhibiting a significantly lower adiponectin mRNA level in the WAT compared with the HFD group (*P* < 0⋅05).

### UCPs mRNA expression in the BAT

**Fig. 6.**
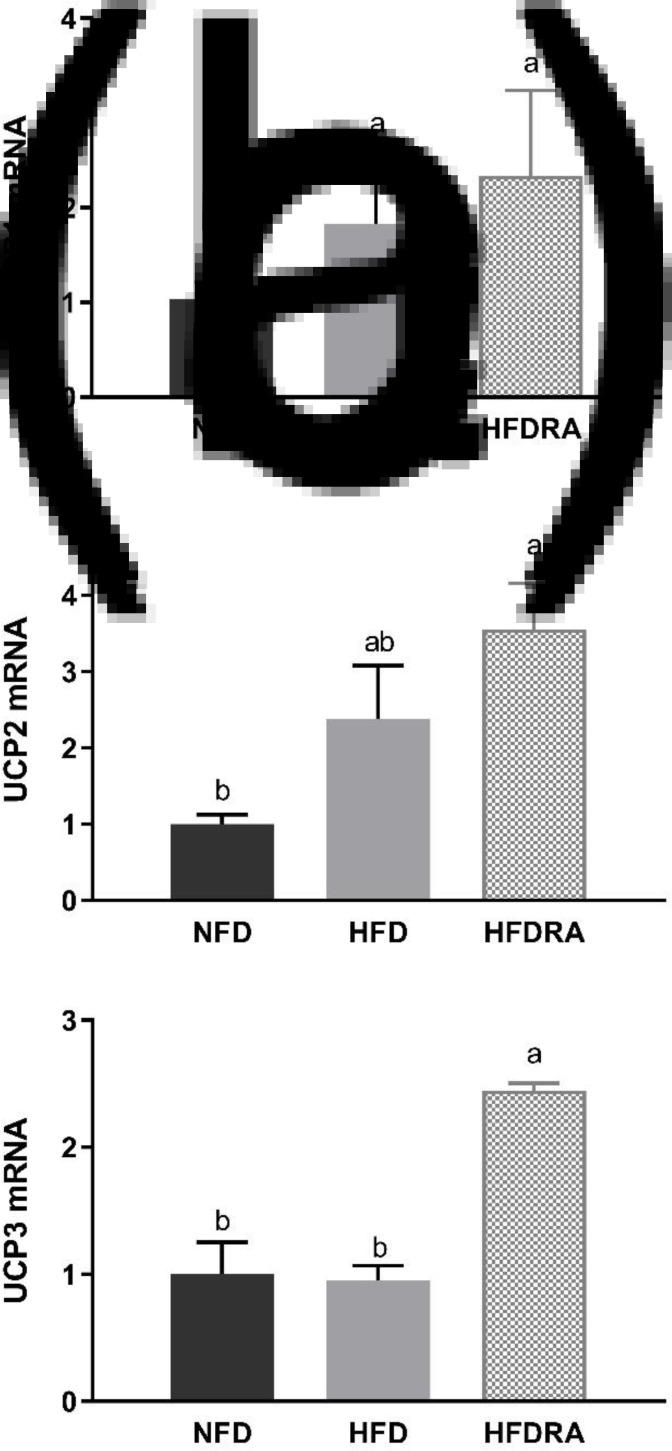
Brown adipose tissue UCP1 (a), UCP2 (b) and UCP3 (c) mRNA expression in rat pups at postnatal day 20. Results were normalised to β-actin mRNA. Bars show means ± sem, *n* 4 per group. One-way ANOVA followed by a Bonferroni post-test was conducted. Different letters indicate statistically significant differences, a > b, *P* < 0⋅05. UCP, uncoupling protein.

The mRNA expression of UCP1, UCP2 and UCP3 was measured using BAT samples from P20 (Fig. 6). No significant difference in UCP1 mRNA expression was observed among the three groups. However, the HFDRA group showed a significantly higher UCP2 mRNA expression than the NFD group (*P* < 0⋅05), while the UCP3 mRNA expression in the HFDRA group was significantly higher than that in the other two groups (*P* < 0⋅05).

## Discussion

To the authors’ knowledge, the present study was the first to determine the effects of RA on the VA status and AT metabolism of neonatal rats in an obesogenic environment.

### RA improved the compromised tissue VA status in neonates caused by maternal HFD consumption

We measured the total retinol concentration in serum and several key organs in the neonatal rats to determine how maternal HFD consumption and RA treatment may affect their VA status, which was the primary aim of the study. Neonatal rats were nursed by dams consuming a marginal VA diet to reduce the transplacental transfer of VA and the concentration of VA in the dams’ colostrum and milk^(32)^. The serum total retinol concentration (Fig. 3(a)) indicated that rat pups in the NFD and the HFD group had an adequate serum VA level according to the criteria for adults (serum retinol: 0⋅7–1⋅75 μmol/l), which is similar to that in newborn human infants as previously reported^(33,34)^. No significant difference in serum total retinol was noted between these two groups, albeit the fact that significant differences in liver and lung total retinol were noted, indicating the well-known homeostatic control of serum VA over a wide range of VA status.

The liver total retinol concentration (Fig. 3(b)) in the NFD group indicated a marginal VA status of the control rat pups (liver VA store: 0⋅035–0⋅07 μmol/g), as expected. At P8 and P14, no significant difference in liver total retinol was noted between the NFD and the HFD group. However, at P20, maternal HFD consumption significantly reduced the neonatal liver VA concentration, bringing it from a marginal to a deficient status (liver VA store < 0⋅035 μmol/g). Previous research in adult Wistar rats showed that the hepatic VA concentration of HFD-fed rats was ~50 % that of the controls^(35)^. Similar results were also noted in obese mice^(18)^. For the first time, we showed in the present study that maternal HFD consumption compromised the liver VA status of the offspring, while liver is the primary storage organ for VA. This could result in significantly reduced VA mobilisation from the liver to the neonatal tissues where VA plays essential roles, such as lung, spleen and brain, and negatively affect developments of these organs during this critical period. Promisingly, the comparison between the HFD and the HFDRA group showed that RA treatment promoted the storage of VA in the liver, as evidenced by a significantly decreased serum total retinol but an increased liver total retinol concentration at P8 and P20. The results are consistent with previous research showing that an acute treatment of RA significantly increased the hepatic gene expression of LRAT, the enzyme that is responsible for the esterification/storage of VA^(36)^.

It is well established that VA is required for postnatal development of the lung, including for alveolar septation, angiogenesis and surfactant synthesis^(10,37)^. Most of the VA in the lungs is in the form of retinyl esters, which can be mobilised to produce RA. A significant accumulation and utilisation of retinyl esters was noted in neonatal lung during the alveolar stage as well as an increase in retinol and RA in lung fibroblasts^(11,38–40)^. The levels of retinoid-binding proteins, RARs and RA synthesising enzymes peak postnatally in the lung^(41)^. RA administered to neonatal rats was found to promote the recovery of the septation process and increase the formation of alveoli, even under conditions of stress^(42,43)^. It was found in the present study that the lung total retinol concentration (Fig. 3(c)) was significantly lower in the HFD pups than in the control group at P8, suggesting that maternal HFD consumption may compromise the lung VA status of the offspring. It is unknown why such an effect was not observed at the later times, but it could be related to the rapid accumulation of retinyl esters in the neonatal lung as pups grew older^(11)^, which might offset the impacts of maternal diet-induced obesity. Indeed, an accumulation of VA in neonatal lung was observed across time in all three groups in the present study, and lung was the only organ in which such an accumulative effect was noted. At all three sampling times, the repeated RA treatments were found to result in a significant 2- to 5-fold increase in the lung VA concentration. The results are consistent with previous findings by others. Wu *et al.* reported that a single dose of RA increased the total retinol concentration, [^3^H]retinol uptake and the mRNA expression of LRAT and STRA6 (stimulated by retinoic acid gene 6, a transmembrane mediator of retinol uptake from circulation into cells) in neonatal rat lung at 6 h after dosing^(23)^. It has been known that maternal overweight/obesity is a significant risk factor for preterm birth, while preterm babies often have low VA status at birth and increased susceptibility to respiratory diseases^(44–46)^. As such, our findings are translatable in that RA could be explored as a promising therapeutic option for those vulnerable newborns in improving their lung VA status, enhancing local RA production and aiding lung development.

It is also worthwhile to emphasise the results on brain VA status (Fig. 3(f)). The neonatal period is characterised by rapid brain development and RA is known to be critical for neurogenesis, neural differentiation and survival, synaptic plasticity, and the formation of new memories and learning^(47–49)^. Previous studies showed that VA deficiency in rodents resulted in reduced performance in memory tasks, which could be restored after VA refeeding^(50–53)^. In the present study, maternal HFD consumption was associated with a significantly lower brain VA status at P8 and P20, while at P14, the opposite trend was noted. The mechanism of the discrepancy is unknown. At P20, RA treatments significantly increased brain VA concentration in the HFD pups and restored it to the concentration in the control group. This is in line with the finding by Hodges *et al.* that maternal VA supplementation significantly increased brain total retinol in rat pups^(54)^. The impacts of obesity on brain VA status and subsequent developmental and functional outcomes warrant further investigation.

The total retinol concentration in WAT and BAT (Fig. 3(d), 3(e)) was also assessed. No significant difference was noted among groups except that the BAT VA concentration was significantly lower in the HFD group than in the control group at P20 and RA treatment showed a trend to increase the concentration. It is plausible that any potentially increased VA uptake by the AT in the HFDRA group was offset by the active utilisation of VA for regulating the tissue development.

### RA reduced the adiposity and modulated the WAT metabolism of suckling rats exposed to maternal HFD consumption

In the present study, maternal HFD consumption during gestation and lactation (up to 20 d) was shown to dramatically increase the adiposity and the BW gain of the neonates (Fig. 2(a), 2(b)). This is consistent with previous findings from others and from our group^(30,55,56)^. The negative effects of maternal obesity or excessive gestational weight gain on the metabolic health of the offspring have been well established. In the present study, we found that repeated RA treatments given orally to the pups of HFD-consuming mothers every 3 d from P5 to P20 exerted a significant effect on reducing their BW gain and the WAT mass. The findings are consistent with our previous study, which showed that VA-supplemented to the maternal HFD significantly reduced the BW and the adiposity of suckling and weanling pups^(30)^. It should be noted that supplementing maternal diet to benefit the health of the offspring can only be utilised in the lactational period, while direct administration of treatments to the offspring, as used in the present study, would allow for a potentially long-lasting effect.

Leptin and adiponectin are two adipokines that are primarily produced by the WAT and are correlated with obesity and metabolic health^(57)^. Leptin can reduce fat storage in adipocytes by inhibiting hunger. Adiponectin plays roles in regulating glucose homeostasis and fatty acid breakdown. In the present study, maternal HFD consumption significantly increased the serum concentrations of leptin and adiponectin as well as their gene expression in the WAT in neonatal rats, while RA treatment exerted a significant reducing effect on the serum concentrations of both and on the WAT gene expression of adiponectin (Figs. 4, 5). The changes may partially be the result of increased WAT in HFD pups and reduced tissue mass by the RA treatment. The findings on serum leptin are consistent with our previous study, in which maternal dietary VA supplementation was also found to decrease the enhanced serum leptin concentration in HFD pups^(30)^. Previous studies in adult rodent models also showed that chronic dietary VA supplementation reduced serum leptin as well as leptin gene expression in WAT^(58,59)^. Acute RA treatment was shown to down-regulate the gene expression of both leptin and adiponectin in WAT in adult rats as well as suppressing leptin gene expression in BAT^(60,61)^. The potential physiological benefits or consequences of RA's regulatory effects on leptin and adiponectin production will need further exploration.

Serum lipid profile was determined in the study. It was noted that the concentration of serum triglycerides was increased in HFD pups compared with NFD pups and was further enhanced in HFDRA pups (Fig. 4(c)). Although the finding was surprising considering that RA reduced the mass of WAT where triglycerides are stored, similar results were reported in rats fed an isotretinoin (13-*cis*-RA)-supplemented diet^(62,63)^. There were also previous case studies reporting that serum triglycerides concentration was increased in patients receiving isotretinoin as acne treatment or following a high dose of VA treatment to patients with pityriasis rubra pilaris^(64,65)^. The development of hypertriglyceridaemia in patients receiving RA-based treatments was discussed by Chen^(66)^. It was noted that RA-induced hypertriglyceridaemia might be due to RA-induced apo CIII expression^(67)^. Apo CIII is considered to be an inhibitor of the activity of lipoprotein lipase, which therefore reduces the clearance of plasma triglycerides^(68,69)^. The effects of RA on the expression of genes invovled in hepatic lipogenesis should be determined in future studies.

### RA influenced the BAT development in suckling rats

BAT is the site for adaptive thermogenesis and is prominent in newborns. In humans, it is gradually lost with age but may still contain beige adipocytes that can be potentially reactivated. Therefore, BAT retains the capacity to play a significant role in energy metabolism and is a primary target in obesity prevention and treatment^(70)^. Our previous study applying maternal dietary VA supplementation showed that maternal consumption of HFD significantly reduced BAT mass while VA supplementation restored the mass^(30)^. However, in the present study, maternal HFD consumption increased the BAT mass in neonates, and oral RA did not exert any effect on the mass (Fig. 2(c)). The discrepancy needs further investigation. However, the finding from the present study that RA treatment significantly reduced the neonatal WAT mass but did not exert the reducing effect on BAT is encouraging.

Moreover, it was found that RA significantly increased the mRNA expression of UCP2 and UCP3 in the BAT of HFD rat pups, although that of UCP1 was not altered (Fig. 6). UCP1 is the inner mitochondrial membrane protein that is responsible for adaptive thermogenesis in BAT^(58)^. In adult rodent models, dietary VA or RA treatment was shown to induce the expression of UCP1^(28,29,71–73)^. In contrast, no change in BAT UCP1 expression was observed in maternal VA-supplemented rat pups in our previous study nor in RA-treated pups in the present study^(30)^. UCP2 and UCP3 genes were cloned in 1997 and the encoded proteins have a high sequence homology to UCP1, but their roles in adaptive thermogenesis and energy metabolism are controversial^(73)^. Compared with the extensive research on UCP1, little is known on the effects of VA or RA on UCP2 and UCP3 expression. Bonet *et al.* reported that acute RA treatment increased BAT UCP2 expression in adult obese mice, which is consistent with our finding^(74)^. The same author group found that RA exerted no significant effect on BAT UCP3 expression, while we found that repeated RA significantly induced its expression in the suckling rats^(58)^. UCP3 was reported to play an important role in regulating the generation of ROS and reducing the oxidative pressure on the respiratory chain^(75)^. Whether our result of increased UCP3 expression in RA-treated BAT reflects an enhanced oxidative stress brought by RA or a protective mechanism induced by RA to maintain the redox balance in HFD rats warrants further investigation.

### Strengths, implications and limitations

The present study has several strengths and accompanying implications. First, maternal diets with a marginal VA level were used to resemble the VA status of at-risk newborns in parts of the developing world or in low-birth-weight infants in the United States^(76)^. Although maternal overweight/obesity is more prevalent in high-income countries, it has also become increasingly prevalent in lower-income countries, including the areas where VA deficiency is a significant nutritional problem in women of childbearing age. A study published in 2014 reported that over half of reproductive-aged women in urban Mauritania are overweight or obese, and the prevalence in urban areas of Kenya, Ghana, Niger, Sierra Leone, Tanzania and Zimbabwe is approaching 50 %^(77)^. Therefore, maternal overweight/obesity may pose a further risk on VA status and the healthy development of infants in those areas which has already been compromised by maternal VA deficiency. As such, our research could be translatable in informing clinical research in both high- and lower-income countries. Secondly, repeated doses of RA were administered for a potentially long-lasting effect. RA was reported in previous studies to have a transient activity in regulating VA homeostatic genes, possibly due to its high turnover rate^(78)^. A single dose of RA was shown to up-regulate the mRNA expression of STRA6, LRAT and CYP26A1 in neonatal lung at 6 h after dose administration, but the effect declined at 12 h^(23)^. In the present study, rat pups euthanised at P8, P14 and P20 received 2, 4 and 6 doses of RA, respectively, and the last dose was given 6 h prior to their euthanisation. Comparing results at those sampling times, it was noted that instead of exerting a cumulative effect, repeated RA treatments every 3 d showed a maintaining effect on most outcomes. Thirdly, collecting data at multiple times provided a dynamic view of neonatal growth and VA status during lactation. Age-related changes in VA status have been reported. Specifically, the VA content of extrahepatic tissues (e.g. lung, heart and brain) have been found to increase with age in rat pups^(79)^.

A few limitations should also be noted. Maternal rats in the HFD groups were provided with the HFD from gestational day 5, which resulted in a relatively short induction period of maternal obesity. A longer induction period during pre-gestation is warranted to better understand the impacts of maternal obesity on the metabolism and development of the offspring. In addition, due to the nature of a neonatal model, the amounts of tissue samples were very limited, which did not allow for more analysis at cellular and molecular levels, and several measures could only be conducted on samples from P14 or P20. Lastly, the sex of neonatal rats was not controlled, but the number of male and female rats was close, which could be a good representation of the general population.

## Conclusion and future directions

To conclude, using a maternal-neonatal rat model, we found that maternal HFD consumption during gestation and lactation posed a significantly negative impact on the BW, adiposity and VA status of the neonatal offspring. Repeated oral RA treatments during the suckling period significantly reduced BW gain and WAT mass, modulated adipokine levels, and improved VA status in key organs of the neonates. Results on the lung VA status were particularly encouraging, considering the critical role of VA in postnatal lung development. Further analysis at the cellular level will be done to fully understand how RA regulates VA metabolism and AT development in neonates exposed to an obesogenic environment. Pre-clinical studies with a longer duration and adopting multiple RA dosages are also needed to elucidate the long-term and dose-dependent effects of RA and to determine the optimal and safe dose.
